# Educational achievement of children with selected major congenital anomalies and associated factors: a Finnish registry-based study

**DOI:** 10.1093/eurpub/ckad149

**Published:** 2023-08-18

**Authors:** Zahra Roustaei, Anna Heino, Sonja Kiuru-Kuhlefelt, Joan K Morris, Svetlana V Glinianaia, Ester Garne, Maria Loane, Judith Rankin, Mika Gissler

**Affiliations:** Department of Health Sciences, University of Helsinki, Helsinki, Finland; Department of Knowledge Brokers, THL Finnish Institute for Health and Welfare, Helsinki, Finland; Department of Knowledge Brokers, THL Finnish Institute for Health and Welfare, Helsinki, Finland; Population Health Research Institute, St George's, University of London, London, UK; Population Health Sciences Institute, Newcastle University, Newcastle upon Tyne, UK; Paediatric Department, Hospital Lillebælt, Kolding, Denmark; Faculty of Life and Health Sciences, Ulster University, Belfast, UK; Population Health Sciences Institute, Newcastle University, Newcastle upon Tyne, UK; Department of Knowledge Brokers, THL Finnish Institute for Health and Welfare, Helsinki, Finland; Academic Primary Health Care Centre, Region Stockholm, Stockholm, Sweden; Department of Molecular Medicine and Surgery, Karolinska Institute, Stockholm, Sweden

## Abstract

**Background:**

Children with major congenital anomalies may be at risk of poor educational outcomes. We aimed to evaluate the educational achievement of children born with major congenital anomalies compared with children without major congenital anomalies in relation to sociodemographic factors.

**Methods:**

We performed a registry-based study including 401 544 children in Finland, graduates of the compulsory school who applied to secondary education. We used health data from the Finnish Register of Congenital Malformations for children born from 1995 to 2002 linked with education data from the Finnish Ministry of Education and Culture. We used generalized linear regression to compare the mean grade differences of children with specific major congenital anomalies and ‘All anomalies’ subgroup (major congenital anomalies, chromosomal syndromes, and multiple anomalies) with reference children.

**Results:**

Children with major congenital anomalies were less likely to apply for further education than reference children (88.0% vs. 96.8%; odds ratio = 4.13; 95% confidence interval, 3.92–4.36). For most non-chromosomal congenital anomalies, children born with congenital anomalies had similar educational achievement to the reference children. For the ‘All anomalies’ subgroup, children with congenital anomalies had lower educational achievement than reference children. Among children with congenital anomalies, male sex, lower maternal educational levels and younger maternal age were associated with lower educational achievement.

**Conclusions:**

For children applying to further education, most non-chromosomal congenital anomalies were not associated with lower educational achievement. Nevertheless, efforts are needed to improve educational achievement in children with major congenital anomalies associated with maternal sociodemographic background.

## Introduction

More than 130 000 children born in Europe annually have a major congenital anomaly.^1^ Over the past several decades, advances in prenatal care and diagnosis, neonatal and paediatric care, early surgical interventions, more safe anaesthesia, new surgical techniques, and medical therapies have improved the survival of children with congenital anomalies beyond infancy in Europe.^2–5^

Despite the considerable improvement in the survival of children with congenital anomalies, there is little evidence on the association between major congenital anomalies and academic performance.^6^ Earlier studies have typically assessed congenital heart defects (CHDs),^7^^,^^8^ single congenital anomalies such as spina bifida^9^ or oral clefts.^10–12^ A recent systematic review and meta-analysis summarized the international evidence on academic achievement of children with congenital anomalies and concluded that selected congenital anomalies were associated with poorer academic achievement.^6^ However, there are few population-based studies on the academic outcomes of children with specific congenital anomalies.^6^ Furthermore, the effects of maternal socioeconomic circumstances and associated factors on child cognitive development have been well established in the general population.^13^^,^^14^ Studies have found that maternal education was associated with educational and developmental outcomes at school in children with CHDs.^8^^,^^15^ Nevertheless, there is little evidence on how sociodemographic factors are linked with educational outcomes among children with major congenital anomalies.

The educational trajectory impacts employability^16^ as well as future mental and physical health outcomes.^17^ Quantifying the academic performance of children with major congenital anomalies and exploring factors associated with educational achievement using high-quality data can serve to guide educational and health interventions.

Therefore, we aimed to investigate the educational achievement of children born with major congenital anomalies compared with children without congenital anomalies (reference children) at the time of graduation from compulsory school in Finland and to determine whether educational achievement is associated with sociodemographic factors. The study was part of the European collaborative project EUROlinkCAT.^1^^,^^18^

## Methods

### Data sources and study population

In this registry-based study, we used health data from the Finnish Register of Congenital Malformations (FRCM), linked with national education data from the Finnish Ministry of Education and Culture (FMEC). The FRCM (established in 1963) contains information on children with major congenital anomalies, including major structural and chromosomal anomalies and minor malformations if notified. Finland is a member of the European network of population-based registries for the surveillance of congenital anomalies (EUROCAT). The FRCM actively collects national data from multiple data sources, and all major congenital anomalies are coded based on the extended version of the *International Classification of Diseases*, Ninth Revision, Clinical Modification (ICD-9-CM) and from the cohort born in 2014, also with the *International Classification of Diseases* External Tenth Revision (ICD-10) codes. The coverage and quality of the FRCM have been estimated to be good since 1993.^19^ We used women’s and children’s unique personal identification numbers (PIN) for data linkages between FRCM and FMEC.

### Exposure: selected major congenital anomalies

EUROCAT adopts the World Health Organization (WHO) definition of major congenital anomalies, as structural changes that have significant medical, social or cosmetic effects on individual, and typically require medical intervention.^20^ In this study, cases were defined as all infants with a major congenital anomaly, as defined in EUROCAT Guide 1.4 (updated version 22 November 2021),^20^ who were alive at ≥23 weeks of gestation between 1 January 1995 and 31 December 2002, alive at age 16 years by 2018 (the last year of available education data at the time of linkage on 12 May 2021), registered as living in Finland at age 16 years, and applied for further education (i.e. general upper secondary education or vocational education and training, including voluntary basic education). The study reference population was all live-born children of the same age and born in the same region as the cases with no major congenital anomaly recorded in the FRCM and no congenital anomaly code in the hospital discharge register. This resulted in an analytical sample of a total of 401 544 children (Supplementary figure S1).

We limited the EUROCAT list of subgroups for structural congenital anomalies to be included in this study to more common isolated congenital anomalies (live birth prevalence ≥1 per 10 000) where children were likely to undergo surgical intervention in early childhood. We have also included the ‘All anomalies’ subgroup that includes children with major structural congenital anomalies, chromosomal syndromes, and multiple congenital anomalies (Supplementary table S1).

### Outcome: educational achievement

Every child who is a permanent resident in Finland is obligated to attend compulsory education. Compulsory education begins the calendar year a child turns seven and ends when the child has completed the basic education syllabus or when 10 years have passed from the start of their compulsory education. Basic education may include an extra voluntary year of additional studies (year 10). At the end of the comprehensive school, each young person who would like to continue in further education must apply for post-comprehensive school education. National individual-level education data are collected and stored by the FMEC for those students who applied to further education, i.e. general upper secondary education or vocational education and training, including voluntary basic education (10th grade).^21^

As there are no national standardized tests in Finland grades at the end of the compulsory school (at approximately 16 years of age) were used to assess educational achievement in children with congenital anomalies compared with reference children.

We used grade means for all school subjects, means for all mandatory and all elective subjects, and means for the following mandatory and optional subjects: native language, mathematics, all foreign languages, science (biology, physics, chemistry combined), social science (philosophy, history, social studies, religion and geography combined) and artistic and practical subjects (arts, crafts, home economics and music combined).

### Definitions

We defined the following diagnoses as severe CHD: common arterial truncus, transposition of great arteries, single ventricle, atrioventricular septal defect, tetralogy of Fallot, pulmonary valve atresia, tricuspid atresia and stenosis, Ebstein anomaly, aortic valve atresia/stenosis, mitral valve anomalies, hypoplastic left heart, coarctation of aorta, aortic atresia/interrupted aortic arch, total anomalous pulmonary venous return (Supplementary table S1). This definition follows the EUROCAT 1.4 guide, except for the hypoplastic right heart and double outlet right ventricle with no ICD-9-CM codes in FRCM from 1995 to 2002.

An isolated congenital anomaly was defined as a structural anomaly in one organ system only or as part of a known sequence. Multiple congenital anomalies were defined as multiple unrelated anomalies across separate anomaly groups. The gestational age was estimated from the date of the last menstrual period and based on first- or second-trimester ultrasonography measurements and categorized as below 32, 32–36 and 37 weeks or more (reference). We classified maternal age as below 20 years, 20–29 (reference), 30–34 and 35 years or more. We classified maternal education into three groups: primary education (9 years or less), secondary education (10–12 years), and the group of post-secondary (13–14 years) and tertiary education (15 years or more) (reference).

### Statistical analysis

We used the chi-square test to compare differences in sociodemographic characteristics of children with congenital anomalies and reference children (child’s sex, birth year, gestational age, maternal age and maternal education).

We calculated unadjusted and adjusted differences in the grade means and 95% confidence intervals (CIs) for the school subjects among children with congenital anomalies and reference children using generalized linear models. We adjusted for baseline confounders including maternal age, child’s sex, maternal education and differences in time periods. Gestational age has been shown to be correlated with educational outcomes^22^; therefore, we have included gestational age in the models.

We estimated the association between sociodemographic factors available in the database and mean grade differences for the ‘All anomalies’ subgroup, using simple and multiple linear regression. As educational achievement may differ in subgroup analysis, we assessed whether there were interactions between maternal age and education, and education and child’s sex by adding the interaction terms in linear regression models. The calculation included the mean grade of children with congenital anomalies across categories of sociodemographic variable in question, minus the mean grade of the reference group of the variable. Negative values of interaction analyses indicated that the sociodemographic variable in question was associated with lower mean grade differences than the reference group while accounting for interaction terms in the models.

The statistical analysis of Finnish data was performed using SAS software (SAS Enterprise guide 7.1; SAS Institute Inc., Cary, NC, USA).

### Ethics approval

Studies using registry-based information without contact to the registered persons do not require ethical approval in Finland. THL (THL/1031/6.02.00/2018), Statistics Finland (TK-53-1195-18) and the National Board of Education (OPH-381-2019) gave their permission to use their administrative health data in this study.

## Results

Of a total of 421 731 children born alive at ≥23 weeks of gestation from 1995 to 2002, 416 105 children were eligible for linkage with education data after exclusion of deaths before age 10 years, those lost to follow-up, and those with incomplete/incorrect PIN: 13 782 children with congenital anomalies and 402 323 reference children. Among these, individual-level national educational data were available only for those children who applied to further education: 401 544 children in total: 12 122 (88.0%) children with congenital anomalies and 389 422 (96.8%) reference children (Supplementary figure S1, table 1).

**Table 1:** The number and the percentage of reference children and children with congenital anomalies by anomaly subgroup who applied to further education

Children’s group	Total number	Applied for further education number (%)	ORs for not applying for further education (95% CI)
Reference children	402,323	389,422 (96.8)	1.00
All anomalies	13,782	12,122 (88.0)	4.13 (3.92–4.36)
Isolated anomalies			
Spina Bifida	57	45 (78.9)	8.05 (4.26–15.22)
Hydrocephalus	66	49 (74.2)	10.47 (6.03–18.19)
CHD	4125	3944 (95.6)	1.39 (1.19–1.61)
Severe CHD	799	761 (95.2)	1.51 (1.09–2.09)
Transposition of great vessels	107	103 (96.3)	1.17 (0.43–3.18)
VSD	2824	2710 (96.0)	1.27 (1.05–1.53)
ASD	1029	973 (94.6)	1.74 (1.33–2.28)
Tetralogy of Fallot	87	81 (93.1)	2.24 (0.98–5.13)
Coarctation of aorta	318	304 (95.6)	1.39 (0.81–2.38)
Cleft lip with or without cleft palate	332	314 (94.6)	1.73 (1.08–2.78)
Cleft palate	406	384 (94.6)	1.73 (1.12–2.66)
Oesophageal atresia with or without tracheo-oesophageal fistula	58	55 (94.8)	1.65 (0.52–5.26)
Ano-rectal atresia and stenosis	75	72 (96.0)	1.26 (0.40–3.99)
Diaphragmatic hernia	23	20 (87.0)	4.53 (1.35–15.24)
Gastroschisis	49	46 (93.9)	1.97 (0.61–6.33)
Multicystic renal dysplasia	98	93 (94.9)	1.62 (0.66–3.99)
Hypospadias	126	121 (96.0)	1.25 (0.51–3.05)
Limb reduction defects	108	100 (92.6)	2.41 (1.18–4.96)
Craniosynostosis	148	141 (95.3)	1.50 (0.70–3.20)
Chromosomal anomalies			
Trisomy 21	490	66 (13.5)	193.92 (149.53–251.49)
Turner syndrome	19	18 (94.7)	1.68 (0.22–12.56)

CAs, congenital anomalies.

### The odds ratios (ORs) of not applying for further education

The OR of not applying for further education was four times higher for children with congenital anomalies compared with reference children (OR = 4.1, 95% CI 3.9–4.4) (table 1). The OR of not applying for further education varied by congenital anomaly, as shown in table 1, being the highest for children with spina bifida (OR = 8.1, 95% CI 4.3–15.2), congenital hydrocephalus (OR = 10.5, 95% CI 6.0–18.2), and in particular for children with trisomy 21 (Down syndrome) (OR = 193.9, 95% CI 149.5–251.5).

### Characteristics of children with congenital anomalies and reference children

The comparison of the characteristics of the study population among children with and without congenital anomalies (table 2) showed a higher percentage of boys among children with congenital anomalies compared with the reference children (54.3% vs. 50.1%). The rate of preterm births (<37 weeks) in children with congenital anomalies was over twice as high as in the reference children (11.3% vs. 5.1%). Mothers of children with congenital anomalies were more likely to be aged 35 years or older. There was no difference in maternal education level between the two groups.

**Table 2:** Sociodemographic characteristics of children with (‘All anomalies’ subgroup) and without (Reference group) major congenital anomalies born from 1995 to 2002 in Finland

**Characteristic, *N* (%)** ^a^	Children with congenital anomalies	Children without congenital anomalies (reference group)
Total (401 544)	12 122 (100.0)	389 422 (100.0)
**Child’s sex**		
Boy	6588 (54.3)	194 988 (50.1)
Girl	5534 (45.7)	194 434 (49.9)
**Gestational age (weeks)**		
<32	231 (1.9)	2082 (0.5)
32–36	1139 (9.4)	17 858 (4.6)
≥37	10 752 (88.7)	369 482 (94.9)
**Maternal age (years)**		
<20	317 (2.6)	10 598 (2.7)
20–29	5710 (47.1)	190 274 (48.9)
30–34	3805 (31.4)	120 829 (31.0)
≥35	2290 (18.9)	67 721 (17.4)
**Maternal education**		
Primary (≤9 years)	1952 (16.1)	62 805 (16.1)
Secondary (10–12 years)	4602 (38.0)	146 397 (37.0)
Post-secondary and tertiary (≥13 years)	5568 (45.9)	180 220 (46.3)
**Child’s year of birth**		
1995	2182 (18.0)	54 629 (14.0)
1996	1420 (11.7)	51 457 (13.2)
1997	1541 (12.7)	49 656 (12.8)
1998	1495 (12.3)	47 682 (12.2)
1999	1414 (11.7)	48 465 (12.4)
2000	1452 (12.0)	47 500 (12.2)
2001	1320 (10.9)	46 920 (12.0)
2002	1298 (10.7)	43 113 (11.1)

a Data presented as number and percentage. Children with congenital anomalies were those who applied to further education.

### Educational achievement at the end of compulsory school

Overall, most children with major congenital anomalies who applied to further education had negligible differences in grade means (table 3, Supplementary table S2). Nevertheless, for the ‘All anomalies’ subgroup, children with congenital anomalies had lower grade means for all school subjects in the adjusted analysis, except for native language, than the reference group (table 3).

**Table 3:** Mean grade differences with 95% CI between children with selected major congenital anomalies (CAs) and reference children

School subject	Number of children with CAs	Number of reference children	Difference in grade means and 95% CI	
			Unadjusted	Adjusted^a^
**All anomalies**				
All subjects	12 122	389 422	−0.08 (−0.09 to −0.06)	−0.05 (−0.06 to −0.03)
All mandatory	12 118	389 371	−0.08 (−0.09 to −0.06)	−0.05 (−0.06 to −0.03)
All elective	11 471	372 139	−0.09 (−0.10 to −0.07)	−0.06 (−0.08 to −0.05)
Native language	12 092	389 239	−0.06 (−0.08 to −0.04)	−0.02 (−0.03 to 0.00)
Mathematics	12 098	389 347	−0.07 (−0.10 to −0.05)	−0.05 (−0.07 to −0.02)
Foreign languages	12 054	389 236	−0.08 (−0.10 to −0.05)	−0.04 (−0.06 to −0.02)
Science^b^	12 087	389 358	−0.07 (−0.10 to −0.05)	−0.05 (−0.07 to −0.03)
Social science^c^	12 094	389 370	−0.05 (−0.07 to −0.03)	−0.03 (−0.04 to −0.01)
Artistic and practical subjects^d^	12 113	389 182	−0.09 (−0.11 to −0.08)	−0.06 (−0.07 to −0.05)
**Selected isolated structural CAs**				
**Spina bifida**				
All subjects	45		−0.20 (−0.46 to 0.06)	−0.27 (−0.51 to −0.04)
All mandatory	45		−0.17 (−0.45 to 0.10)	−0.25 (−0.49 to −0.01)
All elective	42		−0.37 (−0.64 to −0.11)	−0.44 (−0.69 to −0.20)
Native language	45		−0.08 (−0.43 to 0.27)	−0.18 (−0.49 to 0.12)
Mathematics	45		−0.25 (−0.66 to 0.15)	−0.32 (−0.70 to 0.07)
Foreign languages	45		−0.08 (−0.44 to 0.28)	−0.15 (−0.48 to 0.18)
Science	44		−0.14 (−0.50 to 0.22)	−0.21 (−0.55 to 0.12)
Social science	45		−0.14 (−0.46 to 0.18)	−0.22 (−0.50 to 0.07)
Artistic and practical subjects	45		−0.31 (−0.53 to −0.10)	−0.38 (−0.57 to −0.20)
**Severe CHD**				
All subjects	761		−0.18 (−0.25 to −0.12)	−0.11 (−0.16 to −0.05)
All mandatory	761		−0.19 (−0.26 to −0.12)	−0.11 (−0.17 to −0.05)
All elective	728		−0.15 (−0.21 to −0.09)	−0.08 (−0.14 to −0.03)
Native language	758		−0.21 (−0.29 to −0.12)	−0.09 (−0.16 to −0.01)
Mathematics	759		−0.18 (−0.27 to −0.08)	−0.12 (−0.22 to −0.03)
Foreign languages	759		−0.27 (−0.35 to −0.18)	−0.18 (−0.26 to −0.10)
Science	759		−0.18 (−0.26 to −0.09)	−0.11 (−0.19 to −0.03)
Social science	759		−0.15 (−0.23 to −0.07)	−0.07 (−0.14 to 0.00)
Artistic and practical subjects	760		−0.16 (−0.21 to −0.11)	−0.08 (−0.12 to −0.03)
**Oesophageal atresia with/without tracheo-oesophageal fistula**				
All subjects	55		−0.28 (−0.52 to −0.04)	−0.25 (−0.46 to −0.04)
All mandatory	55		−0.30 (−0.55 to −0.05)	−0.28 (−0.49 to −0.06)
All elective	55		−0.13 (−0.37 to 0.10)	−0.09 (−0.30 to 0.12)
Native language	55		−0.34 (−0.66 to −0.03)	−0.26 (−0.54 to 0.01)
Mathematics	55		−0.42 (−0.79 to −0.06)	−0.43 (−0.78 to −0.08)
Foreign languages	55		−0.29 (−0.62 to 0.03)	−0.28 (−0.58 to 0.02)
Science	55		−0.35 (−0.67 to −0.03)	−0.36 (−0.65 to −0.06)
Social science	55		−0.28 (−0.57 to 0.00)	−0.27 (−0.53 to −0.01)
Artistic and practical subjects	55		−0.21 (−0.4 to −0.01)	−0.13 (−0.29 to 0.04)
**Coarctation of aorta**				
All subjects	304		−0.17 (−0.27 to −0.07)	−0.10 (−0.19 to −0.01)
All mandatory	304		−0.18 (−0.29 to −0.08)	−0.11 (−0.21 to −0.02)
All elective	289		−0.09 (−0.19 to 0.02)	−0.02 (−0.12 to 0.07)
Native language	302		−0.2 (−0.34 to −0.07)	−0.09 (−0.20 to 0.03)
Mathematics	302		−0.19 (−0.35 to −0.03)	−0.15 (−0.30 to 0.00)
Foreign languages	302		−0.27 (−0.41 to −0.13)	−0.20 (−0.33 to −0.07)
Science	302		−0.2 (−0.33 to −0.06)	−0.14 (−0.27 to −0.01)
Social science	302		−0.15 (−0.27 to −0.02)	−0.07 (−0.18 to 0.04)
Artistic and practical subjects	304		−0.14 (−0.23 to −0.06)	−0.06 (−0.13 to 0.01)

Notes: ‘All anomalies’ subgroup includes children with isolated structural anomalies (about 80% of all anomalies), children with associated major anomalies and chromosomal syndrome. CAs, congenital anomalies. Unadjusted and adjusted difference in grade means using linear regression.

a Adjusted for birth year, child’s sex, gestational age, maternal age, maternal education.

b Mandatory and optional biology, physics, chemistry combined.

c Mandatory and optional philosophy, history, social studies, religion and geography combined.

d Mandatory and optional arts, crafts, home economics and music combined.

Children with severe CHD had lower grade means for all subjects except for social science (table 3). However, for all CHDs, the grade means were similar for many school subjects (Supplementary table S2). For children with spina bifida, the grade means differences for all subjects −0.27 (−0.51 to −0.04), including all mandatory −0.25 (−0.49 to −0.01) and all elective subjects −0.44 (−0.69 to −0.20), were lower in the adjusted analyses (table 3). Despite relatively large differences in grade means for specific subject groups varying between −0.15 and −0.32, they did not reach statistical significance. Similarly, the grade differences for children with oesophageal atresia with/without tracheo-oesophageal fistula were lower for all subjects −0.25 (−0.46 to −0.04), all mandatory subjects −0.28 (−0.49 to −0.06), mathematics −0.43 (−0.78 to −0.08), science −0.36 (−0.65 to −0.06) and social science −0.27 (−0.53 to −0.01) (table 3).

The following congenital anomalies were not associated with lower grade means for the majority of school subjects in the adjusted analyses: hydrocephalus, transposition of great arteries, ventricular septal defect (VSD), tetralogy of Fallot, cleft lip with/without cleft palate, cleft palate, ano-rectal atresia or stenosis, diaphragmatic hernia, gastroschisis, multicystic renal dysplasia, hypospadias, limb reduction defects and craniosynostosis (Supplementary table S2).

### Factors associated with educational achievement

Table 4 shows the association between sociodemographic factors and the mean grade differences among children with congenital anomalies (‘All anomalies’ subgroup). There were sex differences in the educational achievement of children with congenital anomalies, whereby boys had lower grade means than girls (adjusted grade mean difference of −0.56). Grade means for children with congenital anomalies were higher for older mothers (30+ years) and lower for younger mothers (<20 years) compared with the reference group (20–29 years). Furthermore, compared with mothers with post-secondary and tertiary education, children with congenital anomalies born to mothers with primary and secondary educational levels had lower grade means. In both unadjusted and adjusted models, preterm birth (both <32 and 32–36 gestational weeks) was not associated with lower grade means compared with children born at term (≥37 weeks). Later birth year was associated with increased grade means (table 4). Similar results were obtained when grade means were compared among children with or without congenital anomalies by sociodemographic factors (Supplementary figure S2). Furthermore, we found statistically significant interactions between maternal age and maternal education and between maternal education and the child’s sex (*P*_interaction_ < 0.0001) in linear regression models for educational achievement among children with congenital anomalies.

**Table 4:** The association between sociodemographic factors and mean grade differences among children with major congenital anomalies (‘All anomalies’ subgroup)

	Difference in grade means (95% CI)			
	Unadjusted (*n* = 12 122)	Adjusted (*n* = 12 122)	Adjusted plus interaction between maternal age and education (*n* = 12 122)	Adjusted plus interaction between maternal education and child’s sex (*n* = 12 122)
**Child’s sex**				
Girl	1.00 (ref)	1.00 (ref)	1.00 (ref)	1.00 (ref)
Boy	−0.57 (−0.60 to −0.54)	−0.56 (−0.59 to −0.53)	−0.56 (−0.59 to −0.53)	−0.56 (−0.59 to −0.53)
**Gestational age (weeks)**				
<32	−0.05 (−0.16 to 0.07)	−0.03 (−0.13 to 0.08)	−0.03 (−0.14 to 0.07)	−0.03 (−0.13 to 0.07)
32–36	−0.03 (−0.09 to 0.02)	−0.02 (−0.07 to 0.03)	−0.02 (−0.07 to 0.03)	−0.02 (−0.07 to 0.03)
≥37	1.00 (ref)	1.00 (ref)	1.00 (ref)	1.00 (ref)
**Maternal age (years)**				
<20	−0.37 (−0.47 to −0.27)	−0.10 (−0.19 to −0.01)	0.14 (0.03 to 0.23)	−0.10 (−0.19 to −0.01)
20–29	1.00 (ref)	1.00 (ref)	1.00 (ref)	1.00 (ref)
30–34	0.14 (0.11 to 0.18)	0.07 (0.04 to 0.10)	−0.05 (−0.02 to −0.09)	0.07 (0.04 to 0.10)
≥35	0.17 (0.08 to 0.25)	0.16 (0.09 to 0.24)	−0.09 (−0.01 to −0.18)	0.16 (0.09 to 0.24)
**Maternal education**				
Primary (≤9 years)	−0.70 (−0.74 to −0.65)	−0.66 (−0.71 to −0.62)	−1.30 (−1.41 to −1.18)	−0.74 (−0.80 to −0.69)
Secondary (10–12 years)	−0.39 (−0.42 to −0.36)	−0.39 (−0.42 to −0.36)	−0.22 (−0.26 to −0.18)	−0.36 (−0.40 to −0.33)
Post-secondary and tertiary combined (≥13 years)	1.00 (ref)	1.00 (ref)	1.00 (ref)	1.00 (ref)
**Child’s year of birth**				
1995	1.00 (ref)	1.00 (ref)	1.00 (ref)	1.00 (ref)
1996	0.03 (−0.03 to 0.08)	0.02 (−0.03 to 0.08)	0.02 (−0.03 to 0.08)	0.02 (−0.03 to 0.07)
1997	0.04 (−0.02 to 0.10)	0.09 (0.04 to 0.14)	0.08 (0.03 to 0.14)	0.09 (0.03 to 0.14)
1998	0.05 (−0.01 to 0.11)	0.09 (0.04 to 0.14)	0.08 (0.03 to 0.13)	0.08 (0.03 to 0.13)
1999	0.11 (0.05 to 0.17)	0.14 (0.09 to 0.19)	0.13 (0.08 to 0.18)	0.13 (0.08 to 0.19)
2000	0.12 (0.06 to 017)	0.15 (0.09 to 0.20)	0.14 (0.09 to 0.19)	0.14 (0.09 to 0.19)
2001	0.16 (0.10 to 0.22)	0.18 (0.12 to 0.23)	0.17 (0.11 to 0.22)	0.17 (0.12 to 0.23)
2002	0.20 (0.14 to 0.26)	0.21 (0.15 to 0.26)	0.19 (0.14 to 0.25)	0.20 (0.15 to 0.26)

In subsequent secondary analyses, we stratified our analyses by comparing educational achievement among children with isolated and multiple congenital anomalies with or without chromosomal syndromes for selected congenital anomalies with a higher number of children (≥100 in the isolated group) (Supplementary table S3). The grade means were significantly lower among children with multiple congenital anomalies compared with those with isolated anomalies for ‘All anomalies’ −0.06 (−0.09 to −0.02), all CHDs −0.17 (−0.24 to −0.9), VSD −0.20 (−0.30 to −0.10), atrial septal defect (ASD) −0.18 (−0.30 to −0.06) and cleft palate −0.24 (−0.40 to −0.07) in adjusted models. The grade means were not different in children with severe CHD, coarctation of aorta, cleft lip with or without cleft palate, limb reduction defects and craniosynostosis between the isolated and multiple anomalies groups. The pattern of results remained unchanged after excluding chromosomal syndromes from the multiple congenital anomalies group (Supplementary table S3).

## Discussion

### Summary of main findings

In this study, four main findings can be drawn. First, children with all major congenital anomalies and selected isolated congenital anomalies had higher ORs for not applying for further education. Second, among Finnish children who applied to further education, the educational achievement at the end of compulsory school for those with selected isolated congenital anomalies was similar to reference children. Nevertheless, there were variations between children with specific congenital anomalies, with children with severe CHD showing lower educational achievement in fundamental school subjects compared with the reference children. For all anomalies combined, children with congenital anomalies had lower educational achievement than reference children. Third, among children with congenital anomalies, male sex, younger maternal age, and lower maternal education were associated with increased risks of poorer educational achievement. Fourth, the grade means were lower in children with multiple congenital anomalies compared with those with isolated anomalies for ‘All anomalies’ subgroup, and this association varied by specific anomaly subgroup.

### Comparison with previous evidence

In line with previous studies, our findings confirmed that children with severe CHD had lower educational achievement than children without congenital anomalies.^8^^,^^23^ It has been shown that more severe CHD, characterized by inadequate cardiac output, number of surgeries and prolonged hypoxia (beginning *in utero*), were associated with poorer cognitive function and educational outcomes.^24^^,^^25^ Brain abnormalities, including reduced brain volumes and altered cortical measurements, and white matter microstructure in adolescents and young adults with severe CHD, have been identified by magnetic resonance imaging.^26^^,^^27^ These abnormalities were associated with poorer neurocognitive outcomes such as mathematics achievement.^27^ Apart from the adverse effects of hypoxia on cognitive functioning, inattention, social interaction impairment and speech and language disorders have been reported among children with CHD.^7^^,^^24^ Homsy et al. found an excess of protein-damaging *de novo* mutations as shared genetic contributions to CHD and neurodevelopmental disabilities.^28^

Furthermore, the negative association between spina bifida and educational achievement is consistent with that of the previous studies.^6^^,^^29^^,^^30^ However, associations between spina bifida and lower grade means for specific school subjects should be cautiously interpreted because of the low number of cases and wide 95% CIs. It is important to note that 21.1% of children with spina bifida and 25.8% of children with hydrocephalus were not included in the analyses because they did not apply for further education. Although poor educational achievement could be reflected in not applying for further education among these children, data used in this analysis could not reveal the reasons for not applying for further education.

In line with other studies, our results suggested that the educational achievement of children with congenital anomalies is lower for children with younger mothers and lower maternal educational levels.^8^^,^^11^^,^^31^ Other studies also showed that maternal smoking, which is more prevalent among women with lower socioeconomic status,^32^ is associated with a higher risk of congenital anomalies and lower academic outcomes in the general population.^32^^,^^33^ Marino et al. indicated that socioeconomic status could influence neurodevelopmental outcomes more than biological or operative factors.^34^ Statistically significant results of interaction analyses in this study further emphasize the importance of screening and early interventions among children with multiple risk factors.

Our results showed that educational achievement was lower for children with multiple anomalies, depending on the specific anomaly subgroup. For instance, the grade means in children with severe CHD in the isolated group were not different than those for children with multiple anomalies. This may indicate that the severity of CHD plays an important role in educational achievement and is associated with lower grade means in this study. However, among children with CHD, VSD, ASD and cleft palate, the presence of associated anomalies may negatively affect educational outcomes. Wernovsky reported that CHD associated with chromosomal anomalies or multiple congenital anomalies are associated with developmental abnormalities and possibility of academic defects.^35^

### Strengths and limitations

The major strengths of this study are the use of data from a population-based registry with a high predictive value and degree of completeness, no attrition and longitudinal design (from the exposure to the outcome at 16 years of age). The use of registry data reduced the risk of threats from reporting and ascertainment bias. The FRCM is a EUROCAT member following standard coding, classification, and inclusion criteria for congenital anomalies. High successful linkage rates allowed us to link information on a wide range of congenital anomalies with the background information on mothers and births for most of our sample.

Of note, the proportion applying for further education was lower among children with major congenital anomalies compared with the reference children (88% vs. 96.8%) (table 1); and only children who applied to further education could be linked to individual-level education data. For instance, the educational outcomes were not available for 12% of children in ‘All anomalies’ subgroup. For some congenital anomalies, the numbers were too small, and therefore the 95% CIs were too wide to reach statistical significance, despite relatively large differences in grade means, for example, for diaphragmatic hernia and spina bifida (table 3, Supplementary table S3). It is important to note that children in Finland receive relevant educational support and health care services, irrespective of socioeconomic backgrounds, potentially reducing the impact of the congenital anomaly(ies) on educational achievement.^20^ In countries with a lower investment in the education and health of children with congenital anomalies, lower academic outcomes would be expected, especially among children with lower socioeconomic backgrounds.

#### Implications

We evaluated the relationship between congenital anomalies and educational achievement to gain insight into the educational needs of these children, which is helpful in guiding policy and research. Early identification and screening of children for any developmental problems and evaluation and re-evaluation for pre-existing or emerging impairments may allow appropriate interventions.^25^^,^^34^ Continued surveillance and screening are particularly beneficial for children with CHD as the risk of developmental disorders changes over time.^34^

Holm et al. found that parents of children with congenital anomalies from different cultures and settings are commonly concerned about their children's quality of life and cognitive and academic outcomes and request more positive information about what their children can achieve.^36^ Our results showed that with universal access to health care and a supportive education system, children with isolated congenital anomalies could have educational achievement similar to the reference children. It is important that this information is provided during counselling parents after prenatal diagnosis of a congenital anomaly or at postnatal diagnosis.

## Conclusion

Educational achievement was largely unaffected among children with many non-chromosomal congenital anomalies applying to further education. Nevertheless, children with all major congenital anomalies combined were more likely to underperform at the end of compulsory school in Finland than those without congenital anomalies; particularly children with severe CHD, who exhibit poorer educational achievement across several fundamental school subjects. Effective management and educational interventions in children with major congenital anomalies, especially among children with lower maternal socioeconomic status, are likely to positively affect their educational achievement and reduce disparities.

## Supplementary Material

ckad149_Supplementary_Data

## Data Availability

The data underlying this article cannot be shared publicly. The data maintained by the Finnish Institute for Health and Welfare and the National Board of Education used under license for this study. The data are, however, available upon authorization application from the THL to researchers who meet the criteria for access to confidential data. Key points Children with congenital anomalies had a higher risk of not applying for further education. Compared with children without congenital anomalies, most children with major congenital anomalies who applied to further education had negligible differences in educational achievement. Among children with congenital anomalies, male sex, younger maternal age and lower maternal education were associated with poorer educational achievement. From the public health importance, effective interventions among children with major congenital anomalies, while considering the sociodemographic characteristics of their mothers could positively affect educational achievement and reduce disparity.
