# Supplementary material for: Educational achievement of children with selected major congenital anomalies and associated factors: a Finnish registry-based study
Source: Eur J Public Health. 2023 Aug 18;33(6):1027–34. doi: 10.1093/eurpub/ckad149 (PMC10710322; doi:10.1093/eurpub/ckad149)

Table S1: Congenital anomalies included in the EUROlinkCAT study of educational outcomes (based on EUROCAT subgroups of congenital anomalies (August 2016) with exclusions listed in chapters 3.2 and 3.3 in EUROCAT Guide 1.4)

EUROCAT Subgroups	ICD10-BPA	ICD9-BPA†
**All anomalies** *	Q-chapter, D215, D821, D1810^, P350, P351, P371	74, 75, 27910, 2281^, 76076, 76280, 7710, 7711, 77121
**Isolated structural anomalies**		
Spina Bifida	Q05	741
Hydrocephalus	Q03	7423
Congenital Heart Defects (CHD)	Q20-Q26	745, 746, 7470-7474
Severe CHD	Q200, Q203, Q204, Q212, Q213, Q220, Q224, Q225, Q230, Q232, Q233, Q234, Q251, Q252, Q262	74500, 74510, 7452, 7453, 7456, 7461, 7462, 74600, 7463, 7465, 7466, 7467, 7471, 74720, 74742
Transposition of great vessels	Q203	74510
VSD	Q210	7454
ASD	Q211	7455
Tetralogy of Fallot	Q213	7452
Coarctation of aorta	Q251	7471
Cleft lip with or without cleft palate	Q36, Q37	7491, 7492
Cleft palate	Q35	7490
Oesophageal atresia with/ without tracheo-oesophageal fistula	Q390-Q391	75030-75031
Ano-rectal atresia and stenosis	Q420-Q423	75121-75124
Diaphragmatic hernia	Q790	75661
Gastroschisis	Q793	75671
Multicystic renal dysplasia	Q6140, Q6141	75316
Hypospadias	Q54	75260
Limb reduction defects	Q71-Q73	7552-7554
Craniosynostosis	Q750	75600
**Chromosomal anomalies**		
Trisomy 21	Q90	7580
Turner syndrome	Q96	75860, 75861, 75862, 75869

All Anomalies = all cases of congenital anomaly, excluding cases with only minor anomalies as defined in Section 3.2 in Guide 1.4 for cases born post-2005. Cases with more than one anomaly are only counted once in the "All Anomalies" subgroup.

†EUROCAT ICD-9 codes are used with the British Paediatric Association (BPA) extension code: https://eu-rd-platform.jrc.ec.europa.eu/sites/default/files/EUROCAT-ICD9-with-BPA-Extension.pdf

Abbreviations: ASD atrial septal defect; CAs congenital anomalies; CHD congenital heart defect; 95% CI 95% confidence interval; VSD ventricular septal defect.

Table S2: Mean grade differences with 95% confidence intervals (CI) between children with congenital anomalies (CAs) not included in main Table 3 and children without CAs using linear regression models.

Anomaly type and school subject	Number of children with CAs	Difference in grade means (95% CI)	
		Unadjusted model	Adjusted[d] model
**Selected isolated structural CAs**			
**Hydrocephalus**			
All subjects	49	-0.18 (-0.44, 0.06)	-0.11 (-0.33, 0.11)
All mandatory	49	-0.18 (-0.45, 0.07)	-0.10 (-0.33, 0.12)
All elective	44	-0.17 (-0.43, 0.08)	-0.08 (-0.32, 0.14)
Native language	48	-0.02 (-0.35, 0.31)	-0.08 (-0.32, 0.14)
Mathematics	48	-0.06 (-0.46, 0.32)	0.002 (-0.37, 0.37)
Foreign languages	48	0.02 (-0.32, 0.37)	0.09 (-0.22, 0.42)
Science[a]	48	-0.16 (-0.51, 0.17)	-0.09 (-0.42, 0.22)
Social science[b]	48	-0.19 (-0.50, 0.11)	-0.12 (-0.39, 0.16)
Artistic and practical subjects[c]	49	-0.31 (-0.52, -0.10)	-0.23 (-0.40, -0.05)
**All congenital heart defects (CHD)**			
All subjects	3 944	-0.00 (-0.03, 0.03)	-0.02 (-0.04, 0.01)
All mandatory	3 942	-0.00 (-0.03, 0.03)	-0.02 (-0.04, 0.01)
All elective	3 767	-0.02 (-0.05, 0.01)	-0.03 (-0.06, 0.00)
Native language	3 937	0.03 (-0.01, 0.06)	0.01 (-0.02, 0.04)
Mathematics	3 939	-0.02 (-0.06, 0.03)	-0.03 (-0.07, 0.01)
Foreign languages	3 937	0.00 (-0.04, 0.04)	-0.02 (-0.05, 0.02)
Science	3 939	0.00 (-0.04, 0.04)	-0.02 (-0.05, 0.02)
Social science	3 939	0.03 (-0.00, 0.07)	0.01 (-0.02, 0.04)
Artistic and practical subjects	3 942	-0.02 (-0.04, 0.00)	-0.03 (-0.05, -0.01)
**Transposition of great vessels**			
All subjects	103	-0.52 (-0.43, -0.09)	-0.14 (-0.29, 0.009)
All mandatory	103	-0.26 (-0.44, -0.08)	-0.14 (-0.30, 0.01)
All elective	99	-0.25 (-0.43, -0.08)	-0.15 (-0.31, 0.003)
Native language	102	-0.20 (-0.43, 0.02)	-0.02 (-0.22, 0.17)
Mathematics	103	-0.20 (-0.47, 0.06)	-0.11 (-0.37, -0.14)
Foreign languages	103	-0.37 (-0.61, 0.13)	-0.24 (-0.46, -0.02)
Science	103	-0.26 (-0.5, -0.02)	-0.14 (-0.36, 0.06)
Social science	103	-0.22 (-0.43, -0.01)	-0.09 (-0.28, 0.09)
Artistic and practical subjects	103	-0.27 (-0.41, -0.12)	-0.14 (-0.26, -0.02)
**VSD**			
All subjects	2710	0.03 (0.001, 0.07)	0.001 (-0.02, 0.03)
All mandatory	2709	0.04 (0.003, 0.07)	0.003 (-0.02, 0.03)
All elective	2587	0.006 (-0.02, 0.04)	-0.01 (-0.05, 0.01)
Native language	2705	0.08 (0.03, 0.12)	0.03 (-0.002, 0.07)
Mathematics	2706	0.02 (0.03, 0.07)	-0,.01 (-0.06, 0.03)
Foreign languages	2704	0.06 (0.01, 0.11)	0.02 (-0.01, 0.06)
Science	2706	0.03 (-0.009, 0,08)	-0.002 (-0.04, 0.04)
Social science	2706	0.07 (0.02, 0.11)	0.02 (-0.009, 0.06)
Artistic and practical subjects	2709	0.01 (-0.01, 0.03)	-0.01 (-0,04, 0,006)
**Tetralogy of Fallot**			
All subjects	81	-0.15 (-0.35, 0.03)	-0.16 (-0.34, 0.003)
All mandatory	81	-0.14 (-0.35, 0.05)	-0.16 (-0.34, 0.02)
All elective	75	-0.23 (-0.42, -0.03)	-0.24 (-0.42, -0.05)
Native language	81	-0.18 (-0.44, 0.06)	-0.19 (-0.42, 0.02)

Mathematics	81	-0.21 (-0.51, 0.08)	-0.22 (-0.51, 0.05)
Foreign languages	81	-0.18 (-0.45, 0.08)	-0.18 (-0.42, 0.06)
Science	81	-0.15 (-0.41, 0.11)	-0.16 (-0.41, 0.07)
Social science	81	-0.06 (-0.30, 0.17)	-0.07 (-0.28, 0.14)
Artistic and practical subjects	80	-0.16 (-0.32, -0.001)	-0.17 (-0.31, -0.04)
**Cleft lip with or without cleft palate**			
All subjects	314	-0.14 (-0.24, -0.04)	-0.02 (-0.11, 0.05)
All mandatory	314	-0.15 (-0.26, -0.05)	-0.03 (-0.12, 0.05)
All elective	300	-0.11 (-0.21, -0.02)	-0.01 (-0.10, 0.07)
Native language	314	-0.20 (-032, -0,06)	-0.01 (-0.12, 0.10)
Mathematics	314	-0.04 (-0.20, 0.10)	0.03 (-0.10, 0.18)
Foreign languages	313	-0.18 (-0.32, -0.04)	-0.05 (-0.18, 0.06)
Science	313	-0.11 (-0.25, 0.01)	-0.008 (-0.13, 0.11)
Social science	313	-0.16 (-0.28, -0.03)	-0.03 (-0.14, 0.07)
Artistic and practical subjects	314	-0.17 (-0.26, -0.09)	-0.04 (-0.11, 0.02)
**Cleft palate**			
All subjects	384	0.008 (-0.08, 0.09)	-0.06 (-014, 0.01)
All mandatory	383	0.01 (-0.07, 0.11)	-0.05 (-0.13, 0.02)
All elective	365	-0.03 (-0.12, 0.05)	-0.10 (-0.18, -0.02)
Native language	384	0.05 (-0.06, 0.17)	-0.06 (-0.17, 0.03)
Mathematics	384	-0.01 (-0.15, 0.12)	-0.05 (-0.18, 0.07)
Foreign languages	383	0.05 (-0.06, 0.18)	-0.01 (-0.13, 0.09)
Science	384	0.02 (-0.09, 0.14)	-0.03 (-0.14, 0.07)
Social science	384	0.05 (-0.05, 0.16)	-0.01 (-0.11, 0.07)
Artistic and practical subjects	384	-0.02 (-0.09, 0.05)	-0.11 (-0.17, -0.05)
**Ano-rectal atresia or stenosis**			
All subjects	72	-0.20 (-0.41, 0.006)	-0.01 (-0.20, 0.16)
All mandatory	72	-0.21 (-0.42, 0.005)	-0.02 (-0.21, 0,16)
All elective	69	-0.14 (-0.35, 0.06)	-0.003 (-0.19, 0.18)
Native language	72	-0.33 (-0.61, -0.06)	-0.07 (-0.31, 0.16)
Mathematics	72	-0.30 (-0.62, 0.01)	-0.15 (-0.45, 0.15)
Foreign languages	72	-0.19 (-0.47, 0.09)	0.01 (-0.25, 0.27)
Science	72	-0.22 (-0.50, 0.05)	-0.048 (-0.30, 0.21)
Social science	72	-0.21 (-0.47, 0.03)	-0.01 (-0.24, 0.20)
Artistic and practical subjects	72	-0.18 (-0.35, -0.01)	0.001 (-0.14, 0.14)
**Diaphragmatic hernia**			
All subjects	20	-0.25 (-0.65, 0.13)	-0.21 (-0.56, 0.13)
All mandatory	20	-0.24 (-0.66, 0.16)	-0.20 (-0.57, 0.15)
All elective	19	-0.33 (-0.72, 0.05)	-0.31 (-0.67, 0.05)
Native language	20	-0.02 (-0.54, 0.50)	0.05 (-0.40, 0.50)
Mathematics	20	-0.40 (-1.00, 0.21)	-0.37 (-0.95, 0.20)
Foreign languages	20	0.08 (-0.45, 0.62)	0.14 (-0.35, 0.63)
Science	20	-0.33 (-0.86, 0.20)	-0.31 (-0.80, 0.18)
Social science	20	-0.27 (-0.75, 0.20)	-0.24 (-0.67, 0.19)
Artistic and practical subjects	20	-0.30 (-0.63, 0.01)	-0.24 (-0.52, 0.02)
**Gastroschisis**			
All subjects	46	0.05 (-0.20, 0.31)	0.15 (-0.07, 0.38)
All mandatory	46	0.05 (-0.21, 0.32)	0.16 (-0.08, 0.40)
All elective	43	-0.002 (-0.26, 0.26)	0.06 (-0.17, 0.30)
Native language	46	0.14 (-0.20, 0.48)	0.25 (-0.04, 0.55)
Mathematics	46	-0.09 (-0.50, 0.30)	0.03 (-0.34, 0.41)
Foreign languages	46	-0.008 (-0.36, 0.34)	0.16 (-0.16, 0.48)
Science	46	0.11 (-0.23, 0.46)	0.23 (-0.09, 0.56)
Social science	46	0.06 (-0.24, 0.38)	0.18 (-0.10, 0.46)

Artistic and practical subjects	46	0.02 (-0.19, 0.23)	0.07 (-0.10, 0.25)
**Multicystic renal dysplasia**			
All subjects	93	-0.17 (-0.36, 0.007)	-0.11 (-0.27, 0.05)
All mandatory	93	-0.18 (-0.37, 0.01)	-0.11 (-0.28, 0.05)
All elective	85	-0.20 (–0.40, -0.02)	-0.15 (-0.32, 0.01)
Native language	93	-0.17 (-0.41, 0.07)	-0.07 (-0.28, 0.13)
Mathematics	93	-0.17 (-0.45, 0.10)	-0.11 (-0.38, 0.15)
Foreign languages	93	-0.13 (-0.38, 0.12)	-0.06 (-0.30, 0.16)
Science	93	-0.22 (-0.47, 0.02)	-0.16 (-0.40, 0.06)
Social science	93	-0.23 (-0.45, -0.01)	-0.16 (-0.36, 0.03)
Artistic and practical subjects	93	-0.14 (-0.28, 0.01)	-0.07 (-0.20, 0.05)
**Hypospadias**			
All subjects	46	0.05 (-0.20, 0.31)	0.15 (-0.07, 0.38)
All mandatory	46	0.05 (-0.21, 0.32)	0.16 (-0.08, 0,40)
All elective	43	-0.002 (-0.26, 0.26)	0.06 (-0.17, 0.30)
Native language	46	0.14 (-0.20, 0.48)	0.25 (-0.04, 0.54)
Mathematics	46	-0.09 (-0.50, 0.30)	0.03 (-0.34, 0.41)
Foreign languages	46	-0.008 (-0.36, 0.34)	0.16 (-0.16, 0.48)
Science	46	0.11 (-0.23, 0.46)	0.23 (-0.08, 0.56)
Social science	46	0.06 (-0.24, 0.38)	0.18 (-0.10, 0.46)
Artistic and practical subjects	46	0.02 (-0.19, 0.23)	0.07 (-0.11, 0.25)
**Limb reduction defects**			
All subjects	100	-0.11 (-0.30, 0.06)	-0.08 (-0.23, 0.076)
All mandatory	100	-0.13 (-0.31, 0.05)	-0.09 (-0.25, 0.06)
All elective	94	0.03 (-0.13, 0.21)	0.07 (-0.09, 0.23)
Native language	100	-0.20 (-0.42, 0.03)	-0.15 (-0.36, 0.04)
Mathematics	100	-0.30 (-0.57, -0.02)	-0.26 (-0.52, -0.006)
Foreign languages	100	-0.24 (-0.48, 0.001)	-0.19 (-0.41, 0.03)
Science	100	-0.16 (-0.40, 0.07)	-0.12 (-0.34, 0.09)
Social science	100	-0.11 (-0.32, 0.09)	-0.08 (-0.27, 0.11)
Artistic and practical subjects	100	-0.03 (-0.17, 0.11)	-0,003 (-0.12, 0.11)
**Craniosynostosis**			
All subjects	141	-0.29 (-0.44, -0.14)	-0.13 (-0.26, 0.00)
All mandatory	141	-0.29 (-0.45, -0.14)	-0.13 (-0.27, 0.00)
All elective	137	-0.33 (-0.48, -0.19)	-0.20 (-0.33, -0.06)
Native language	141	-0.28 (-0.48, -0.08)	-0.01 (-0.18, 0.16)
Mathematics	141	-0.29 (-0.52, -0.06)*	-0.20 (-0.42, 0.02)
Foreign languages	141	-0.3 (-0.51, -0.10	-0.14 (-0.33, 0.04)
Science	141	-0.3 (-0.50, -0.10)	-0.17 (-0.36, 0.01)
Social science	141	-0.31 (-0.49, -0.13)	-0.14 (-0.30, 0.02)
Artistic and practical subjects	141	-0.32 (-0.44, -0.20)	-0.12 (-0.23, -0.02)

Unadjusted and adjusted difference in grade means using linear regression.

[a] Mandatory and optional biology, physics, chemistry combined.

[b] Mandatory and optional philosophy, history, social studies, religion and geography combined.

[c] Mandatory and optional arts, crafts, home economics and music combined.

[d] Adjusted for birth year, sex, gestational age, maternal age, maternal education.

Abbreviations: CAs congenital anomalies; 95% CI 95% confidence interval; VSD ventricular septal defect.

Table S3: Mean grade differences for children with isolated and multiple congenital anomalies with or without chromosomal syndromes.

CA subgroup*	Multiple CAs		Multiple CAs without chromosomal syndromes		Isolated		Unadjusted Model	P value	Unadjusted Model without chromosomal syndromes	P value	Adjusted Model	P value	Adjusted Model without chromosomal syndromes	P value
	Number	Grade mean	Number	Grade mean	Number	Grade mean	Difference in means (95% CI)		Difference in means (95% CI)		Difference in means (95% CI)		Difference in means (95% CI)	
All anomalies	2 577	7.84	1804	7.85	9 545	7.92	-0.08 (-0.12, -0.04)	<0.001	-0.07 (-0.11, -0.02)	0.004	-0.06 (-0.09, -0.02)	0.0020	-0.04 (-0.08, -0.007)	0.1021
All CHD	610	7.79	415	7.80	3 944	7.98	-0.18 (-0.26, -0.11)	<0.001	-0.17 (-0.26, -0.08)	0.0002	-0.17 (-0.24, -0.9)	<0.001	-0.14 (-0.23, -0.05)	0.0015
Severe CHD	168	7.79	100	7.76	761	7.80	-0.01 (-0.15, 0.14)	0.896	-0.04 (-0.22, 0.14)	0.6821	-0.03 (-0.17, 0.11)	0.6837	-0.05 (-0.23, 0.13)	0.5865
VSD	309	7.78	211	7.78	2 710	8.01	-0.24 (-0.34, -0.13)	<0.001	-0.23 (-0.36, -0.11)	0.0003	-0.20 (-0.30, -0.10)	<0.001	-0.16 (-0.28, -0.04)	0.0092
ASD	228	7.80	149	7.81	973	7.95	-0.14 (-0.27, -0.02)	0.027	-0.14 (-0.29, -0.01)	0.0762	-0.18 (-0.30, -0.06)	0.0041	-0.16 (-0.31, -0.01)	0.0321
Coarctation of aorta	45	7.68	31	7.63	304	7.81	-0.13 (-0.41. 0.15)	0.348	-0.18 (-0.51. 0.15)	0.2929	-0.14 (-0.42. 0.14)	0.3158	-0.12 (-0.45, 0.20)	0.4497
Cleft lip with or without cleft palate	57	7.88	36	7.94	314	7.83	0.05 (-0.20, 0.30)	0.670	0.11 (-0.20, 0.41)	0.4975	0.19 (-0.11, 0.50)	0.2146	0.19 (-0.11, 0.50)	0.2146
Cleft palate	138	7.72	96	7.75	348	7.99	-0.26 (-0.44, -0.09)	0.003	-0.24 (-0.44, -0.03)	0.0246	-0.24 (-0.40, -0.07)	0.0066	-0.21 (-0.40, -0.01)	0.0373
Limb reduction defects	137	7.89	107	7.92	100	7.86	0.03 (-0.18, 0.24)	0.790	0.06 (-0.17, 0.28)	0.6324	0.02 (-0.23, 0.19)	0.8464	0.03 (-0.20, 0.25)	0.8072
Craniosynostosis	43	7.75	33	7.93	141	7.69	0.06 (-0.25, 0.36)	0.716	0.24 (-0.11, 0.58)	0.1730	0.12 (-0.19, 0.43)	0.4401	0.30 (-0.05, 0.64)	0.0890

*Grade means for all school subjects and difference in means (with 95% CI) are presented for ‘All anomalies’ and selected CAs with the number of children ≥100 in the isolated group. Cases with multiple CAs are counted as once in “All anomalies” and once in subgroups to which the anomalies belong.

Abbreviations: ASD atrial septal defect; CHD congenital heart defect; 95% CI 95% confidence interval; CAs congenital anomalies; VSD ventricular septal defect.

Figure S1: Flowchart of selection of study participants.

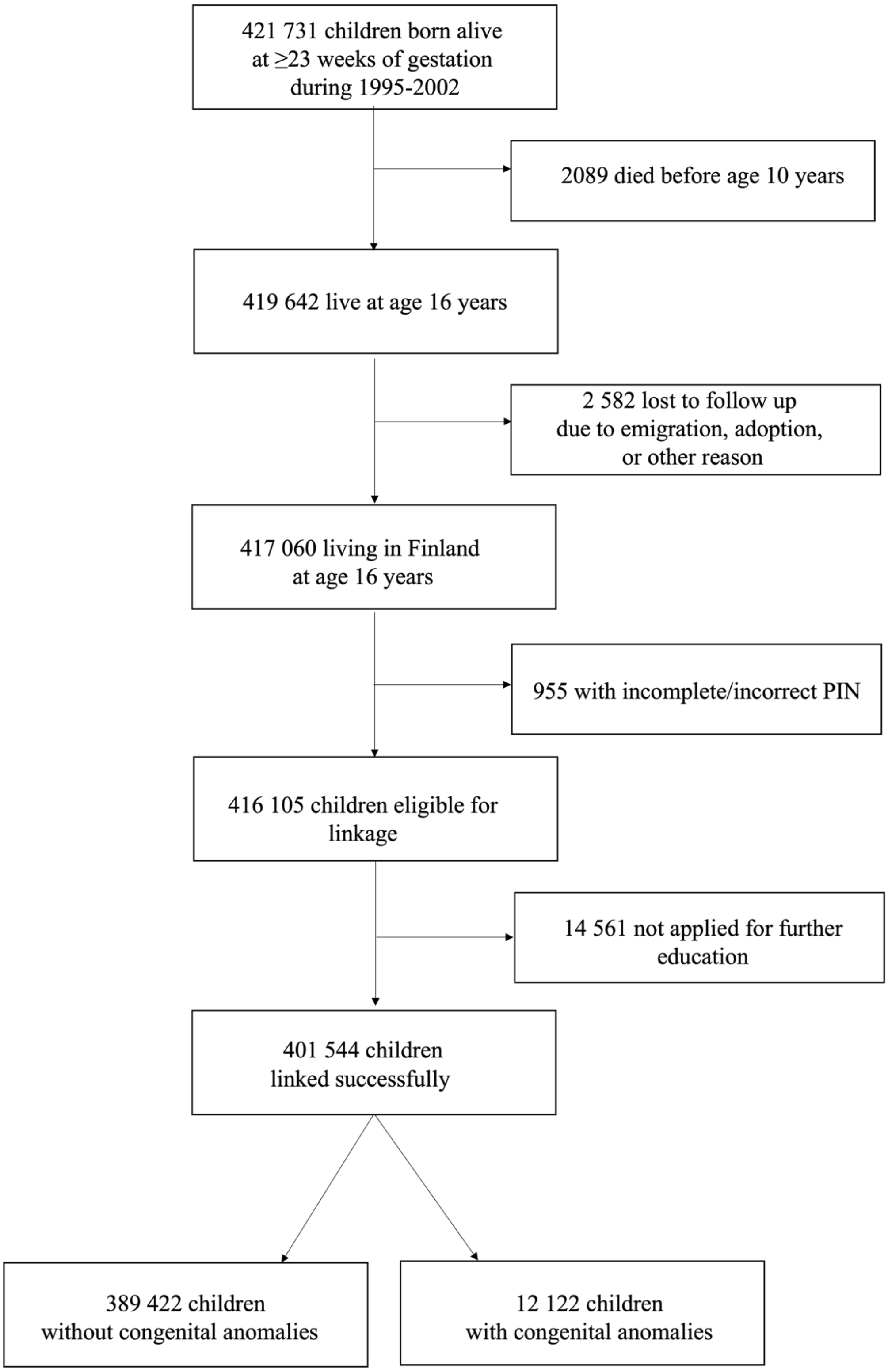

PIN, personal identification number

Figure S2: Grade means at the end of compulsory school among children with major congenital anomalies and children without major congenital anomalies by sociodemographic factors. In Finland, grades at the end of the compulsory school year were scaled from 4 to 10, with 10 being the highest grade.

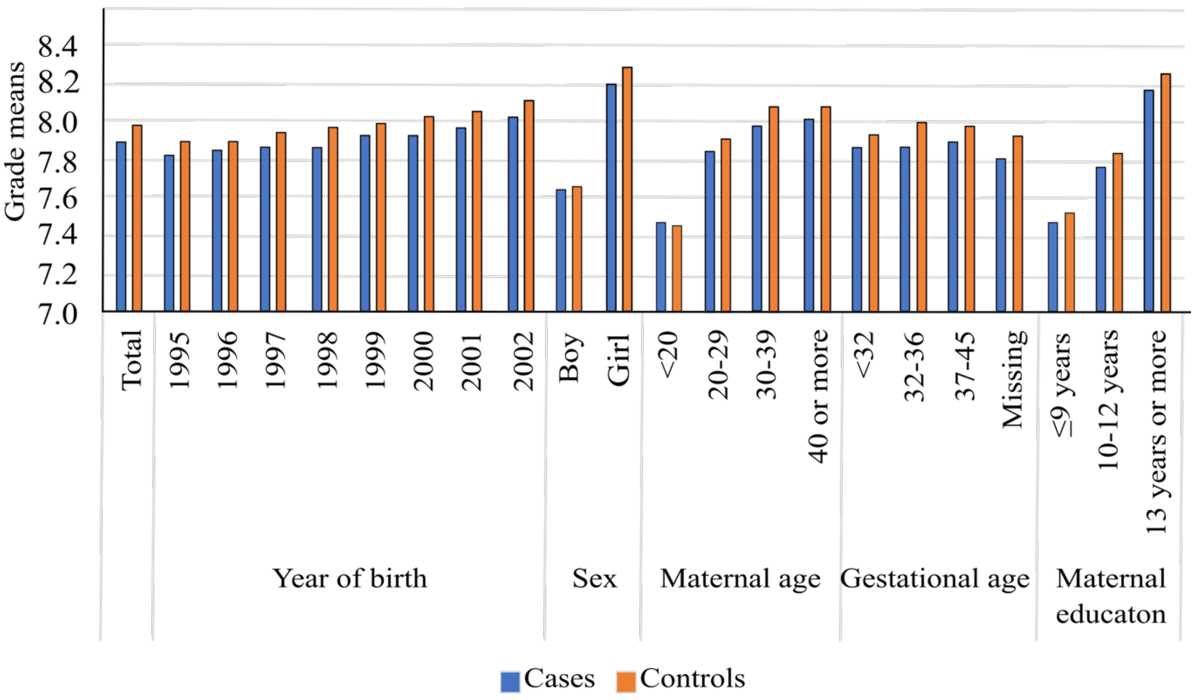

Supplement: ckad149_Supplementary_Data [file ckad149_supplementary_data.pdf]
